# Supplementary material for: Capturing dynamical correlations using implicit neural representations
Source: Nat Commun. 2023 Sep 20;14:5852. doi: 10.1038/s41467-023-41378-4 (PMC10511537; doi:10.1038/s41467-023-41378-4)
Supplement: Supplementary file 1 — Supplementary Information [file 41467_2023_41378_MOESM1_ESM.pdf]

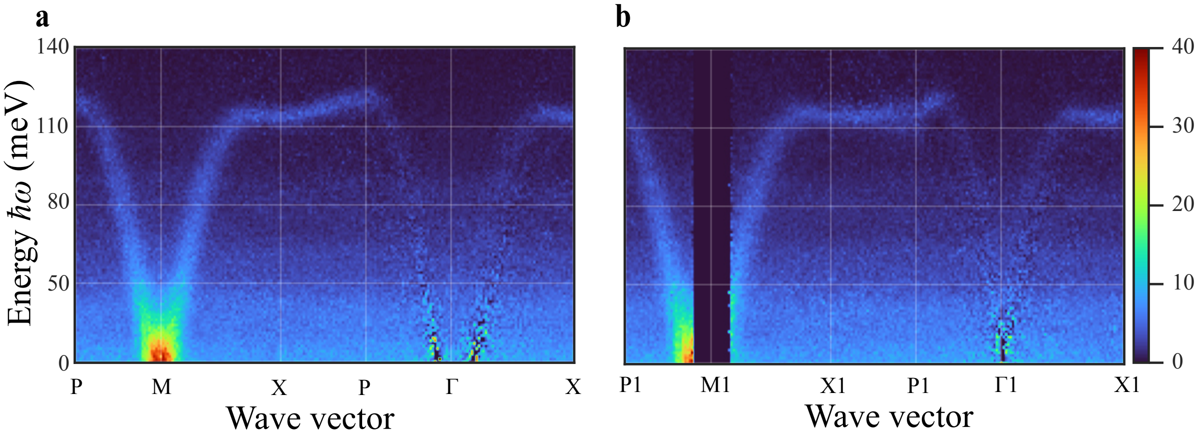

Figure S1. Inelastic neutron scattering dataset without automated background subtraction. Visualization of **a** path 1 and **b** path 2 for inelastic neutron scattering data without automated background subtraction (Fig. S1). Note, path 2 contains a portion of missing data.

a

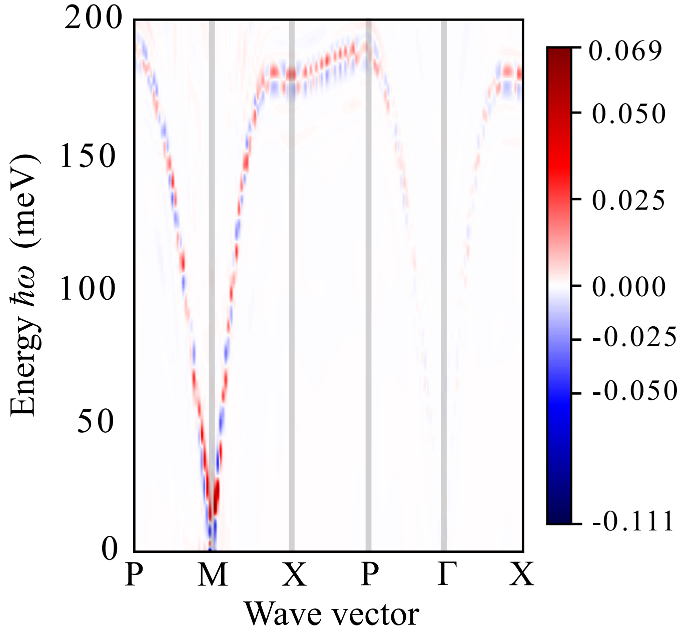

Figure S2. Visualization of the numerical subtraction between LSWT simulation and machine learning forward model prediction. (a) Difference plot with minimum and maximum colorbar limits set at the maximal positive and negative deviation and corresponding to the calculation in Fig. 2. Note, there appears to be some alternating patterns in the difference profile. This is likely due to the discrete choice of momenta simulated with SpinW which are smoothed out by the continuous machine learning prediction. In regions outside of the dispersion curve, the difference is 0 since the model is able to confidently predict that there is no signal.

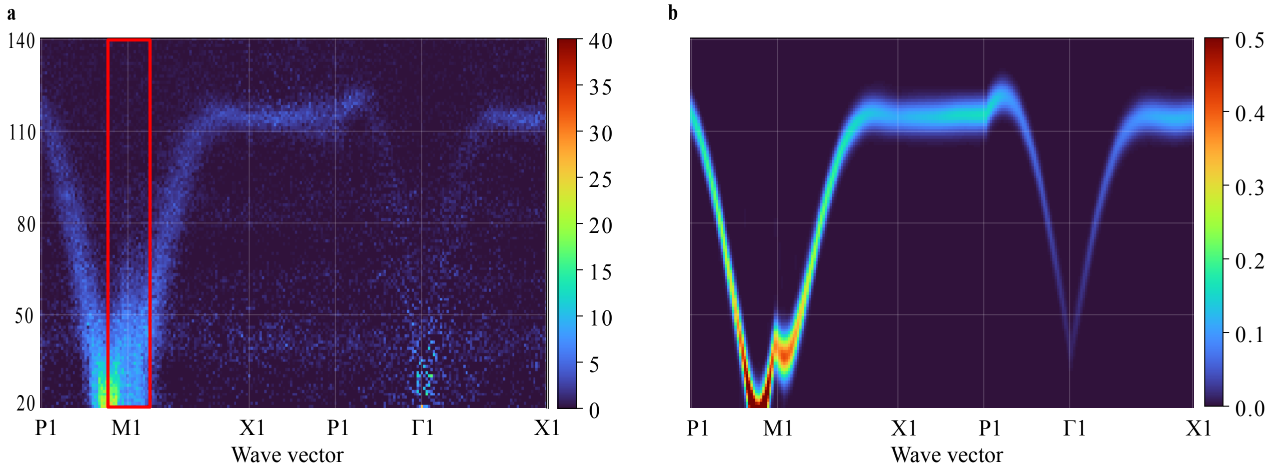

Figure S3. Machine learning forward model accurately predicts scattering profile for a missing region (red rectangle) of path 2 data. For the displayed path there is no data available in the utilized neutron dataset for the missing region of **Q**-space. However, data is available for $(Q_x, Q_y) \mapsto (Q_y, Q_x)$, so for the 2D momenta of the missing path region rotated by 45°. As this compound is assumed to be tetragonal magnetically ordered$S(Q_x, Q_y, \omega) = S(Q_y, Q_x, \omega)$ and thus, the missing region in **Q** can be substituted by the equivalent data with Q_x and Q_y exchanged. The data along the full path with the missing region substituted is depicted in comparison with the result predicted by our forward model. **a** Experimental data with missing region filled in by exchanging Q_x and Q_y. **b** machine learning prediction using only experimental data from path 2 with missing region. Evidently, the machine learning prediction closely models the true experimental data. Note, in this case, the prediction for path 2 only uses the data from path 2. No information from path 1 is utilized in the fitting.

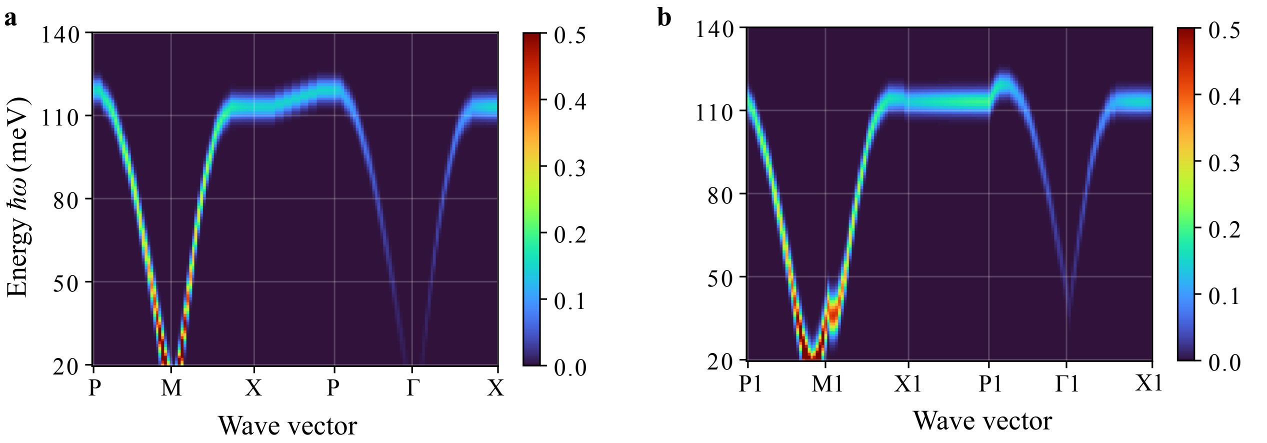

Figure S4. SpinW fitting results for the (J, J_p) parameters when both path 1 and path 2 are used for the fitting. SpinW optimization yielded Hamiltonian parameters $(J, J_p) = (29.15, 1.55)$ meV. Corresponding forward predictions for **a** path 1 and **b** path 2. Relative to the machine learning method, SpinW fitting does not utilize all the available pixel information and instead requires additional peak finding and peak fitting steps.
